# sto-3 is expressed in R4BL/R and R8BL/R, male-specific ray neurons in C. elegans

**DOI:** 10.17912/micropub.biology.000091

**Published:** 2019-03-06

**Authors:** Vladislav Susoy, William Joseph Joyce, Sahand Jamal Rahi

**Affiliations:** 1 Harvard University, Department of Physics, Cambridge, MA, USA; 2 University of Massachusetts Medical School, Neurobiology Department, Worcester, MA, USA; 3 Laboratory of the Physics of Biological Systems, Institute of Physics, École Polytechnique Fédérale de Lausanne (EPFL), CH-1015 Lausanne, Switzerland

**Figure 1:**
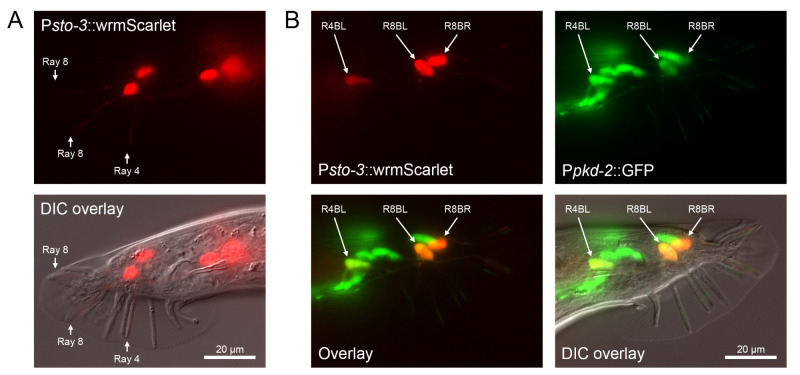
P*sto3*::wrmScarlet reporter is expressed in R4BL/R and R8BL/R ray neurons.

## Description

In *C. elegans* hermaphrodites, *sto-3* promoter has been previously shown to drive gene expression in RIBL/R neurons and in three unidentified non-neuronal cells in the tail (Turek et al., 2016). (A) We have found that in *C. elegans* males, in addition to RIBL/R, two pairs of bilaterally-symmetrical tail neurons show strong P*sto3*::wrmScarlet expression (wrmScarlet is a codon-optimized version of mScarlet (El Mouridi et al., 2017)). These neurons send their processes to rays 4 and 8 of the male tail. In the figure, right lateral aspect is shown; arrows indicate rays containing processes expressing P*sto3*::wrmScarlet. (B) All rays in the male tail are innervated by A and B type neurons (Sulston et al., 1980). To identify which neuron type expresses the *sto-3* reporter, we have crossed P*sto3*::wrmScarlet transgenic males with a P*pkd-2*::GFP reporter strain MT11318, which expresses GFP in the B-type neurons of rays 1-5, 7-9 and not in the A-type neurons (Barr and Sternberg, 1999). In the F1 cross-progeny males, which also carried the *ceh-30*(n3714) mutation in the background, P*sto-3*::wrmScarlet expression is colocalized with P*pkd-2*::GFP for both pairs of ray neurons expressing P*sto-3*::wrmScarlet. This indicates that *sto-3* is expressed in the R4BL/R and R8BL/R ray neurons. Left lateral aspect; arrows point at the cell bodies of P*sto3*::wrmScarlet-expressing neurons.

## Reagents

*zfEx898*[P*sto-3*::wrmScarlet + lin-15(+)]; lin-15 (n765ts) X. P*sto-3*::wrmScarlet transcriptional fusion. The plasmid was made by cloning 971 bp promoter region of *sto-3* into a wrmScarlet-unc-54 3’UTR vector with a pUC19 vector backbone.

Strains: QW1876.

## References

[R1] Barr MM, Sternberg PW (1999). A polycystic kidney-disease gene homologue required for male mating behaviour in C. elegans.. Nature.

[R2] El Mouridi S, Lecroisey C, Tardy P, Mercier M, Leclercq-Blondel A, Zariohi N, Boulin T (2017). Reliable CRISPR/Cas9 Genome Engineering in *Caenorhabditis elegans* Using a Single Efficient sgRNA and an Easily Recognizable Phenotype.. G3 (Bethesda).

[R3] Sulston JE, Albertson DG, Thomson JN (1980). The Caenorhabditis elegans male: postembryonic development of nongonadal structures.. Dev Biol.

[R4] Turek M, Besseling J, Spies JP, König S, Bringmann H (2016). Sleep-active neuron specification and sleep induction require FLP-11 neuropeptides to systemically induce sleep.. Elife.

